# Extensive In Vivo Resilience of Persistent *Salmonella*

**DOI:** 10.1371/journal.pone.0042007

**Published:** 2012-07-24

**Authors:** Somedutta Barat, Benjamin Steeb, Alain Mazé, Dirk Bumann

**Affiliations:** Focal Area Infection Biology, Biozentrum, University of Basel, Basel, Switzerland; University of Osnabrueck, Germany

## Abstract

Chronic infections caused by persistent pathogens represent an important health problem. Here, we establish a simple practical mouse *Salmonella* infection model for identifying bacterial maintenance functions that are essential for persistency. In this model, a substantial fraction of *Salmonella* survived even several days of treatment with a potent fluoroquinolone antibiotic indicating stringency of the model. Evaluation of twelve metabolic defects revealed dramatically different requirements for *Salmonella* during persistency as compared to acute infections. Disrupted synthesis of unsaturated/cyclopropane fatty acids was the only defect that resulted in rapid *Salmonella* clearance suggesting that this pathway might contain suitable targets for antimicrobial chemotherapy of chronic infection.

## Introduction

Persistent pathogens represent a major problem for control of infectious diseases [1]. Extensive drug tolerance of persisters to all available antimicrobials often leads to treatment failures and relapse. Persistent pathogens may adopt a non-replicating dormant stage with no requirement for macromolecular synthesis comprising most current antimicrobial targets [2]. As a consequence, such dormant stages are tolerant to most antibiotics. In addition, low metabolic activity during dormancy might minimize vulnerability to perturbation. In fact, it remains unclear if dormant persisters have any essential maintenance requirements for survival that could provide opportunities for eradication through antimicrobial chemotherapy.

Various in vitro models have been used as an approximation of chronic infection with dormant persisters. Data obtained with these models revealed differential perturbation effects depending on the particular model and the respective pathogen [3], [4], [5], [6], [7], [8]. As an example, proton motive force-driven ATP synthesis has been shown to be essential for *Mycobacterium tuberculosis* survival in a hypoxia in vitro model [9]. Indeed, inhibition of ATP synthase accelerates mycobacterial eradication in patients [10]. On the other hand, diminishing ATP levels can actually promote *E. coli* in vitro persister formation [1]. Additional in vivo models could help to compare persister maintenance requirements under relevant conditions. However, except for *Mycobacterium tuberculosis*, practical in vivo persistency models are largely lacking.

*Salmonella enterica* can cause diarrhea or systemic disease called typhoid/paratyphoid fever. A substantial fraction of systemically infected individuals develops asymptomatic chronic infection [11], [12]. In many cases, *Salmonella* persists in biofilms on gallstones but persisting *Salmonella* have also been detected in liver [13] and lymph nodes [14]. Surgical removal of gallstones is required for successful treatment of *Salmonella* in gallstones biofilms, while extended treatment with potent fluoroquinolone antibiotics is recommended for treatment of chronic *Salmonella* tissue colonization [15].

In genetically resistant mice, *Salmonella* cause an acute infection with exponential *Salmonella* proliferation. However, after *Salmonella* peak colonization and partial clearance *Salmonella* persist at low levels and this can cause relapses [14]. Genetic screens have identified some factors that might support chronic *Salmonella* survival in this model [12]. However, *Salmonella* mutants defective for genes relevant during the initial acute phase would be lost early on without reaching persistency. This problem could be circumvented using inducible gene cassettes but this is impractical for testing many candidate genes.

In this study, we used a simple chronic mouse *Salmonella* infection model in which a substantial *Salmonella* subpopulation survived without previous exponential proliferation. Interestingly, *Salmonella* survived even prolonged treatment with a fluoroquinolone antibiotic thus mimicking treatment failures. In this stringent in vivo model, almost all tested *Salmonella* activities were dispensable confirming extensive resilience of persistent pathogens against perturbation. On the other hand, the data also revealed a few novel candidate targets that could be explored for their suitability to control chronic infections.

## Results

### Persistency Model using *Salmonella purA ssaGH*

**Figure 1:**
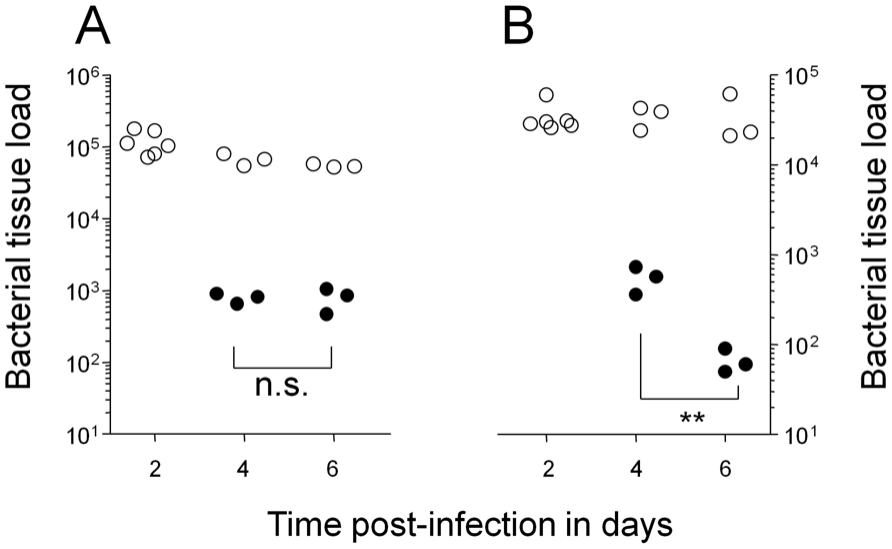
Colonization kinetics of *Salmonella enterica serovar Typhimurium purA ssaGH* in systemically infected BALB/c mice. Data are shown for spleen (**A**) and liver (**B**) of individual untreated mice (open circles), and mice that were treated from day two post infection with enrofloxacin (filled circles). Statistical significance of clearance at day 6 compared to day 4 were determined by t-test of log-transformed data (**, *P*<0.01; n.s., not significant).

Wildytpe *Salmonella* SL1344 grew exponentially in spleen of infected genetically susceptible BALB/c mice (Figure S1A). To generate a practical *Salmonella* persistency model, we constructed a *Salmonella* SL1344 derivative that survived but largely failed to proliferate in systemically infected mice. Specifically, we combined two mutations that had previously been shown to impair *Salmonella* in vivo growth: *purA* which blocks adenosine biosynthesis [16], and *ssaGH* which inactivates the SPI-2 (*Salmonella* pathogenicity island 2)-associated type three secretion system required for intracellular *Salmonella* growth and virulence [17]. Both *purA* and SPI-2 mutations have previously been shown to result in long-term persistence with minimal acute virulence, but our initial characterization revealed some in vivo proliferation of the individual mutants after i.v. administration (Figure S1B). In contrast, the double mutant *Salmonella purA ssaGH* was initially largely cleared from spleen and liver (Figure S1C) consistent with early killing during acute salmonellosis [18], but maintained largely constant bacterial tissue loads thereafter (Fig. 1A, B) suggesting limited net growth.

To determine the suitability of this model for evaluating antimicrobial targets during persistency, we treated infected mice with the antibiotic enrofloxacin. This antibiotic belongs to the fluoroquinolone class, which is uniquely effective against non-growing bacteria in vitro [19], and the treatment of choice for human persistent salmonellosis although effective therapy might require several weeks of treatment [15]. Enrofloxacin has been shown to be well absorbed after oral administration, with penetration into all tissues [20]. Indeed, enrofloxacin is the most effective drug in the mouse typhoid fever where it diminishes wildtype *Salmonella* loads in spleen and liver to levels below the detection threshold within one to two days of treatment although relapses occur unless treatment is continued for several days indicating some residual *Salmonella* persistence [21], [22]. In our persistency model, the same enrofloxacin treatment initially diminished spleen loads of *Salmonella purA ssaGH*, but in contrast to previous findings for wildtype *Salmonella*, a substantial surviving subpopulation of *Salmonella purA ssaGH* stabilized within two days and remained clearly detectable during at least four days of treatment (Fig. 1A). Liver loads continuously decreased during prolonged treatment suggesting somewhat different *Salmonella* physiological states and/or differential pharmacokinetics in the two host tissues. We determined MIC (minimal inhibitory concentration) values of the inocculum and ten clones recovered from spleen and liver of two different mice after four days of enrofloxacin treatment. All clones were enrofloxacin sensitive with the same MIC value of 0.06 mg l^−1^ indicating that *Salmonella* persisted because of partial tolerance or limited antibiotic availability, but not emergence of resistant mutants. The substantially increased persistence of *Salmonella purA ssaGH* during enrofloxacin treatment indicated that our model offered a practical approach to study treatment failures during persistency. Enrofloxacin efficacy also provided a suitable benchmark for potential new *Salmonella* persistency targets.

### *Salmonella* Defects with Minor Persistency Phenotypes

**Figure 2:**
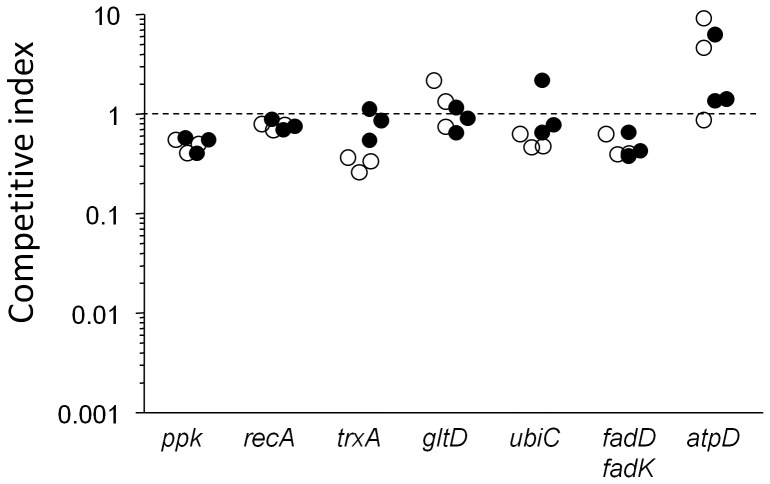
Competitive indices (CI) of various *Salmonella* mutants vs. the parental *Salmonella purA ssaGH* strain in infected spleen (open circles) and liver (filled circles). Data are shown for individual mice at day seven post infection. A competitive index of 1 indicates equal colonization capabilities of mutant and parental strains. Statistical significance was determined by t-test of log-transformed data. Spleen colonization of mutants *ppk, recA, trxA, ubiC*, and *fadD fadK* was significantly lower compared to the parental strain (*P*<0.05). Liver colonization of mutants *ppk, recA,* and *fadD fadK* was significantly lower compared to the parental strain (*P*<0.05).

Only a small number of *Salmonella* genes are absolutely essential for *Salmonella* survival and growth in host tissues during acute salmonellosis [23]. Some of these genes might also be relevant for *Salmonella* persistency. To test this hypothesis we transduced 12 mutations into the parental *Salmonella purA ssaGH* strain, and determined persistence capabilities of the resulting strains in competitive infections with mixtures with the parental strain. At day 7 post infections, most strains had small colonization defects compared to the parental strain as indicated by competitive indices that were close to 1. These data suggested that most tested genes had only minor impact on *Salmonella* persistency in our model despite their crucial importance during acute infections (Fig. 2).

As an example, *ubiC* encoding chorismate lyase is required for ubiquinone biosynthesis. During acute infection, *Salmonella ubiC* is completely cleared from infected mice within one day indicating absolute essentiality [23]. In striking contrast, *Salmonella purA ssaGH ubiC* survived at high levels indicating dispensability of ubiquinone-mediated oxidative respiration during persistency. Similarly, functional ATPase is essential for acute *Salmonella* virulence [24], but we found it to be fully dispensable during persistency. This was in striking contrast to various *Mycobacterium tuberculosis* models that suggest ATPase to be a particularly attractive target for this pathogen [9], [10]. Another case with strikingly different relevance in acute [25] vs. persistent *Salmonella* infections was *recA* involved in DNA repair. More expectedly, *trxA* encoding a thioredoxin essential for SPI-2 function [26], had no detectable role in *Salmonella purA ssaGH* presumably because SPI-2 was already inactive in this strain.

Polyphosphate biosynthesis or fatty acid degradation were known to be largely dispensable during acute infection but had some role in other chronic *Salmonella* infection models [27], [28]. However, in our stringent model both activities had weak effects indicating their dispensability for persistency. All these negative results suggested a severely limited number of suitable targets for control of persistent *Salmonella* infections.

### *Salmonella* Defects with Moderate Persistency Phenotypes

**Figure 3:**
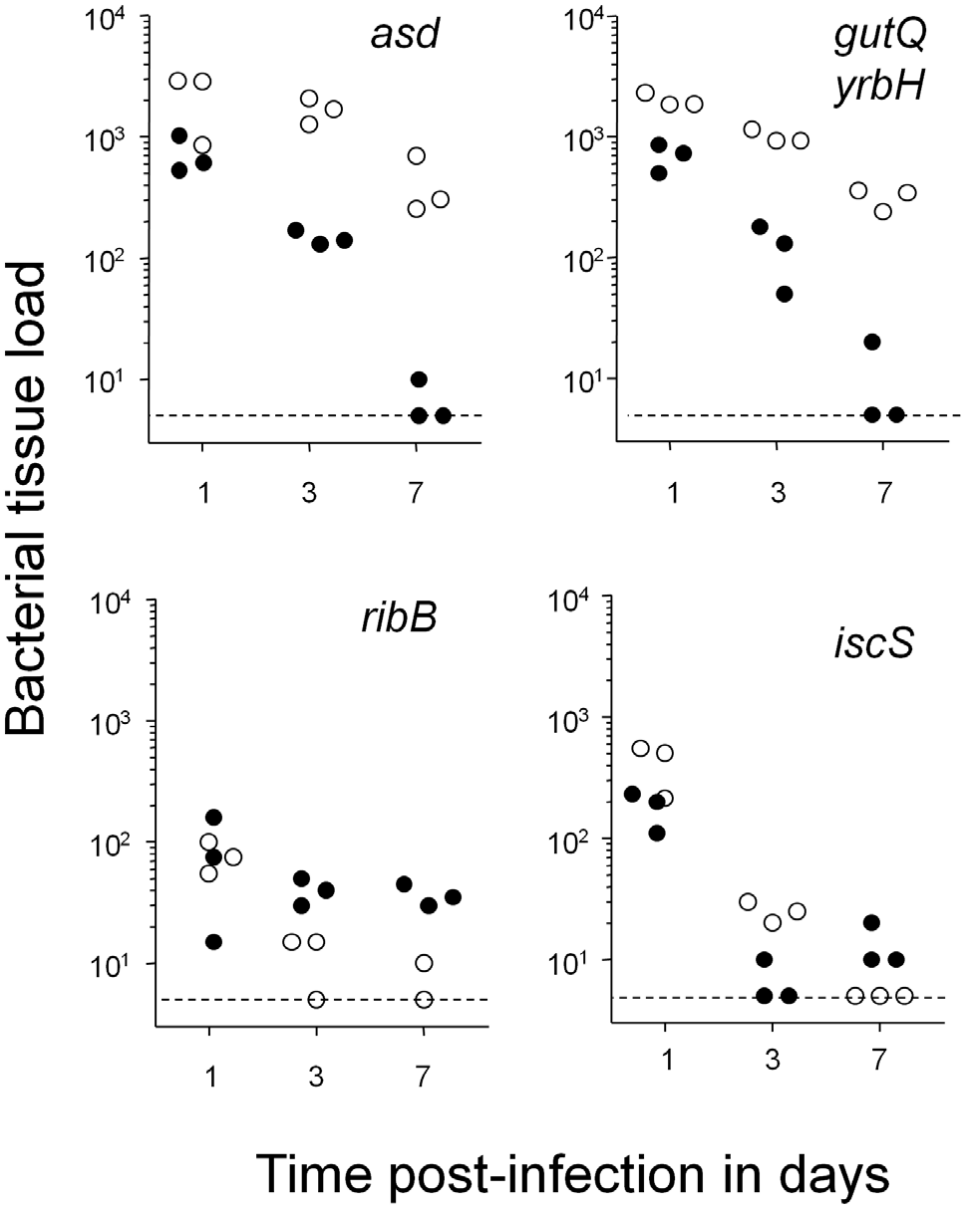
Colonization kinetics of four compromised mutants in spleen (open circles) and liver (filled circles). Small residual colonization levels after seven days of infection suggested that all shown genes contributed to *Salmonella* survival but were not absolutely essential. Statistical significance of clearance at day 7 compared to day 1 in spleen was determined by t-test of log-transformed data (***, *P*<0.001).

In contrast to all these cases, two mutations, *asd* and *gutQ yrbH*, showed moderate phenotypes in our model (Fig. 3). *asd* encoding aspartate semialdehyde dehydrogenase is required for biosynthesis of the cell-wall peptidoglycan component diaminopimelic acid. A *Salmonella asd* strain spontaneously lyses in vitro and is completely cleared within one day from systemically infected mice [23]. However, *Salmonella purA ssaGH asd* was only partially cleared during the first day post infection which might reflect residual proliferation of some *Salmonella* and/or difficulties in establishing a suitable systemic niche [18]. Thereafter, this strain persisted at slowly declining levels in spleen. This could reflect non-essentiality of cell-wall synthesis for non-growing bacteria [19]. In contrast, liver loads rapidly declined suggesting a substantial fraction of *Salmonella purA ssaGH* with active cell-wall turnover/growth in liver. Similarly, *Salmonella purA ssaGH gutQ yrbH* that required supplementation with the lipopolysacharide precursor arabinose-5-phosphate to grow in vitro [29] and was highly attenuated during acute infections (our unpublished data), maintained high levels in spleen but was cleared from liver suggesting limited lipopolysaccharide demands during *Salmonella* persistency. Both genes thus were unsuitable as targets.

Two additional mutants had very severe colonization defects but still maintained stable small loads indicating non-essentiality for seven day persistence. *Salmonella purA ssaGH ribB* defective for 3,4-dihydroxy-2-butanone 4-phosphate synthase which is involved in riboflavin biosynthesis, was cleared within one day post infection to very low levels in both spleen and liver, but stabilized thereafter particularly in liver. This might reflect differential availability of host riboflavin supplementation in these two tissues. Importantly, these data showed that *Salmonella* with defective riboflavin biosynthesis can survive in vivo for extended periods. Another strain that was rapidly cleared from spleen had a defect in *iscS* encoding cysteine desulfurase involved in repair of iron-sulfur clusters and tRNA modification [30]. This mutant also dropped to very low loads in liver but still maintained detectable loads at seven days post infection.

### β-ketoacyl-ACP Synthase I Essentiality for *Salmonella* Persistency

**Figure 4:**
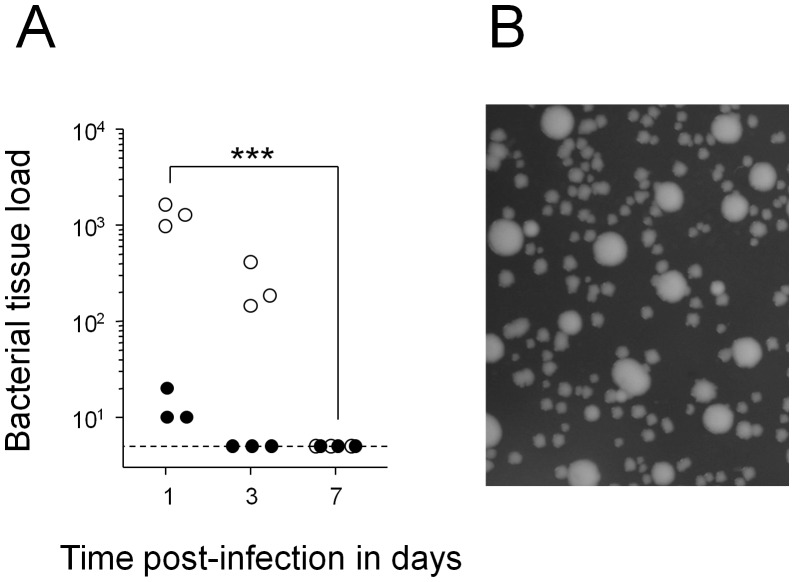
Clearance of *Salmonella purA ssaGH fabB* from infected mice. A) Colonization kinetics in spleen (open circles) and liver (filled circles). Similar results were obtained in three independent experiments. Statistical significance of clearance at day 7 compared to day 1 were determined by t-test of log-transformed data (***, *P*<0.001; **, *P*<0.01; *, *P*<0.05; n.s., not significant). B) Heterogeneity of colony size on agar plates. Similar data were obtained for two independent in vitro cultures and five independent ex vivo cultures.

Finally, there was a single mutant with a more promising phenotype (Fig. 4A). *Salmonella purA ssaGH fabB* defective for β-ketoacyl-ACP synthase I required for biosynthesis of unsaturated fatty acids and cyclopropane fatty acids, was progressively cleared from both liver and spleen. During clearance, residual *Salmonella purA ssaGH fabB* were recovered from mice mostly as small-colony variants. Withdrawal of fatty acid supplementation in vitro similarly enriched small-colony variants of this strain (Fig. 4B), suggesting that reduced growth and metabolism might enhance survival of this mutant when external fatty acids are unavailable. However, even small-colony variants were rapidly cleared from mouse tissues. Small colony variants usually reflect decreased growth rate which can be caused by diverse *Salmonella* defects such as dimished respiratory activity [31]. Elucidation of the actual mechanisms that caused our SCV’s was difficult because small-colony variants of *Salmonella fabB* generated in vivo or in vitro quickly reverted to fast growth upon sub-culturing in presence of oleic acid supplementation.

To test the suitability of this target for antimicrobial chemotherapy, we administered thiolactomycin [32], a slow onset inhibitor of β-ketoacyl-ACP synthase I that is effective in mouse infection models with extracellular pathogens [33]. However, safe doses did not diminish *Salmonella purA ssaGH* loads in spleen (data not shown). This could reflect the low target affinity of this inhibitor and/or poor delivery to *Salmonella* that persist intracellularly in infected macrophages [14], [21].

## Discussion

Chronic infections represent a major health problem. Eradication often requires long-term treatment that causes compliance problems, facilitates resistance development, and often fails to prevent relapse. Many chronic infections are likely to be caused by persistent pathogens in a dormant state with minimal cellular and metabolic activities. In fact, it remains unclear if such dormancy has any basal maintenance requirements that could be targeted for therapy. Various in vitro and in vivo models have been established to determine requirements of persistent pathogens. However, results depend on the particular model and it remains unclear how well these models mimic relevant conditions during chronic infections. It is likely that even within one infected host tissue, various microenvironments exist that might induce distinct forms of persistency [34], [35].

Here we established a simple *Salmonella* mouse infection model in which *Salmonella* with dual metabolic and virulence defects persisted at constant tissue loads without an initial acute infection that hampers functional analysis in a more natural *Salmonella* infection in genetically resistant mice [12]. Indeed, in this model a substantial fraction of such *Salmonella* reached a non-proliferating state with minimal cell wall turnover within one day post infection in spleen, and a substantial *Salmonella* subpopulation even survived chronic treatment with a fluoroquinolone. This was surprising since the same treatment diminishes wildtype *Salmonella* loads in spleen to non-detectable levels [21], [22], and since fluoroquinolones are the most potent, but still only partially effective antibiotic to eradicate persistent salmonellosis [15] and non-growing bacteria in general [2], [19]. These data suggested that our model represented a stringent test for identifying targets that might be useful in clinically relevant settings. On the other hand, the emergence of small-colony variants of a *fabB* mutant suggested that in this model persistent *Salmonella* still had some metabolic activities that could be diminished to relax residual maintenance requirements.

*Salmonella* in liver remained sensitive to fluoroquinolone treatment and required continuous de novo cell wall synthesis. These data suggested that despite purine auxotrophy and inactivity of the SPI-2 type III secretion system, liver microenvironments might permit residual *Salmonella* proliferation in this tissue. Liver colonization was therefore less suitable as readout for *Salmonella* maintenance requirements during persistency. On the other hand, distinct *Salmonella* microenvironments in this organ provided complementary information for target evaluation. As an example, liver seemed to provide conditions that enable at least partial survival of *Salmonella* mutants defective for riboflavin biosynthesis or repair of iron-sulfur clusters, in contrast to conditions in spleen. Antimicrobial chemotherapy should eradicate *Salmonella* from all host organs including liver suggesting that the corresponding targets might be unsuitable.

The *Salmonella* metabolic network contains more than 1200 different enzymes that could all represent potential antimicrobial targets. However, only a very small number of these enzymes are sufficiently important for *Salmonella* physiology to qualify as potentially suitable targets to control acute infections [23]. Interestingly, the data from this study suggested that almost all of these targets might be unsuitable to treat persistent infections indicating strikingly different *Salmonella* requirements for survival as compared to growth in host tissues. Activities that were absolutely essential during acute infection, but dispensable during persistency, include cell wall synthesis, ubiquinone-dependent aerobic respiration, proton motive force-dependent ATP synthesis, translational accuracy, DNA repair, and thioredoxin-mediated redox balance. Dispensability of PMF-driven ATP synthesis highlighted the stringency of our model but might also reflect differences between *Salmonella* and other pathogens. In addition, activities that play important roles in other persistency models including fatty acid degradation and polyphosphate storage were also dispensable in our model. These data indicate that *Salmonella purA ssaGH* had limited requirements for extended in vivo survival.

We found only a single defect, inactive biosynthesis of unsaturated fatty acids and cyclopropane fatty acids that resulted in clearance to non-detectable tissue loads within a few days. Interestingly, clearance kinetics for defective mutants were faster compared to the best current antimicrobial drug enrofloxacin for chronic salmonellosis suggesting that the corresponding targets could potentially help to improve treatment of such disease. It is possible that defective fatty acid biosynthesis could result in accumulation of toxic intermediates although such toxic intermediates have not yet been described in the respective pathway and the mutant grows normally in vitro if supplemented with oleic acid. Flux-Balance Analysis [36] of a genome-scale metabolic model [37] predicted additional expected essential genes in the fatty acid biosynthesis pathway (*accA, accB, accC, accD, acpP, fabA, fabD, fabG, fabI*) but no other pathways reflecting redundancy in providing required precursors such as malonyl-CoA, NADPH, and NADH.

Unsaturated fatty acids and their derivatives cyclopropane fatty acids together comprise about one-half the *Salmonella* fatty acid content [38]. Essentiality of de novo synthesis could suggest continuous internal turnover, damage, and or loss to the environment. Damage/loss of membranes has previously been proposed as a potential strategy to control persisters [39]. Reactive oxygen species can readily damage mammalian polyunsaturated fatty acids, but bacterial unsaturated fatty acids that usually contain only a single double bond are refractory to oxidative damage [40]. Alternatively, membranes could also be lost by shedding outer membrane vesicles [41]. On the other hand, continuous synthesis of another outer membrane component, lipopolysaccharide may not be needed for *Salmonella* persistence based on the slow clearance of *Salmonella purA ssaGH gutQ yrbH* from infected spleen (Fig. 3). Further studies are needed to clarify the function of de novo fatty acid synthesis and the impact of the host immune response on fatty acid requirements during *Salmonella* persistency. It is also important to note that host fatty acids (both saturated and unsaturated) might be sufficiently available in other infectious disease models, especially in case of extracellular pathogens [42].

In conclusion, we established a practical, highly stringent in vivo persistency model. Data obtained with this model revealed that key metabolic activities that are essential during acute salmonellosis might be dispensable during persistent *Salmonella* infections. On the other hand, at least some *Salmonella* metabolic activities might be crucial for persistency and the model could help to identify additional requirements in subsequent studies.

## Materials and Methods

### Bacterial Genetics

We used strain *Salmonella enterica* serovar typhimurium SL1344 *hisG xyl* [43] as parental wild type strain. *Salmonella* mutants were constructed by lamda red- recombinase mediated allelic replacement [44] followed by general transduction using phage P22 *int* [45]. Resistance cassettes were flanked with FRT sites for removal using FLP recombinase [44]. All strains were cultivated at 37°C in Lennox LB medium containing 90 µg/ml streptomycin and 50 µg/ml kanamycin, 20 µg/ml chloramphenicol, and/or 100 µg/ml ampicillin, as appropriate. Auxotrophs were supplemented with 40 µg/ml riboflavin (*ribB*), 0.1% oleate (*fabB*), 50 µg/ml diamino pimelic acid (*asd*), 15 µM D-arabinose-5-phosphate/10 µM glucose-6-phosphate (*gutQ yrbH*). Agar plates containing oleate were always freshly prepared and maintained at 37°C to keep oleate homogeneously dispersed. Minimal inhibitory concentrations (MIC) for enrofloxacin were determined as described [46].

### Mouse Infections

All animals were handled in strict accordance with good animal practice and all animal work was approved by local animal care and use committee (license 2239, Kantonales Veterinäramt BS). Eight to 12 weeks old female BALB/c mice were infected intravenously with 10^6^ CFU *Salmonella* from late exponential LB cultures. For some experiments, we administered enrofloxacin (2 mg/ml) in the drinking water beginning two days post infection [21], or thiolactomycin (two doses of 2 mg per mouse). For competitive infections, mutant *Salmonella* carrying different antibiotic resistance cassettes were mixed before administration. The actual bacterial dose was confirmed by plating. At various time intervals post infection, mice were sacrificed, spleen and liver collected aseptically in 1 ml of 0.1% Triton Tx-100, and number of viable bacteria per organ was determined by plating tissue homogenates on appropriate selective media. Competitive indices (CI = output ratio/input ratio) were calculated based on plate counts for inoculum and tissue homogenates collected at seven days post infection.

### In Silico Modeling

To predict additional targets, we used a genome-scale computational *Salmonella in vivo* metabolism model STMv1.1, an updated version of the consensus genome-scale metabolism reconstruction STMv1 [37] (manuscript in preparation). We used production of unsaturated fatty acids as objective function and determined all genes that were predicted to be essential for this function with Flux-Balance Analysis [36] using MatLab and the COBRA toolbox [47].

## Supporting Information

Figure S1 **Colonization kinetics of various** ***Salmonella*** **mutants in spleen (open circles) and liver (closed circles) of systemically infected BABL/c mice.** A) Colonization of wildtype *Salmonella* SL1344 after systemic infection with 350 CFU. B) Colonization of SL1344 *purA* after infection with 1.85×10^6^ CFU and SL1344 *ssaGH* after infection with 1.2×10^6^ CFU. C) Initial colonization of SL1344 *purA ssaGH* after infection with 8.5×10^5^ CFU. Statistical significance of colonization level differences at day 2 and 4 (for data in A), clearance at day 7 compared to day 1 (for data in B), or colonization levels at 24 h compared to 2 h (for data in C) were determined by t-test of log-transformed data (***, *P*<0.001; **, *P*<0.01; *, *P*<0.05).
(TIF)
