# Supplementary figures and images for: Extensive In Vivo Resilience of Persistent Salmonella
Source: PLoS One. 2012 Jul 24;7(7):e42007. doi: 10.1371/journal.pone.0042007 (PMC3404010; doi:10.1371/journal.pone.0042007)

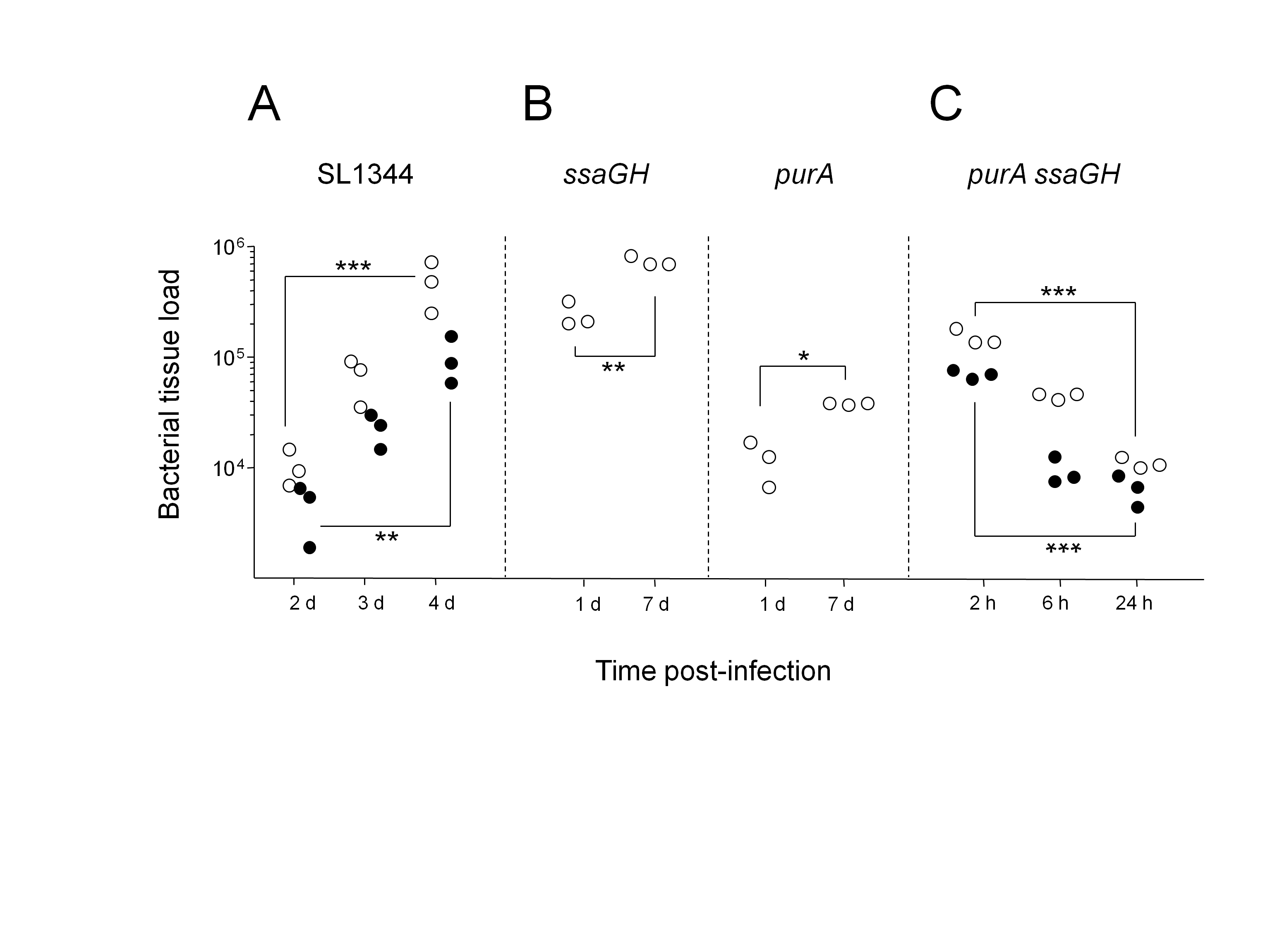

Supplement: Figure S1 — Colonization kinetics of various Salmonella mutants in spleen (open circles) and liver (closed circles) of systemically infected BABL/c mice. A) Colonization of wildtype Salmonella SL1344 after systemic infection with 350 CFU. B) Colonization of SL1344 purA after infection with 1.85×10^6^ CFU and SL1344 ssaGH after infection with 1.2×10^6^ CFU. C) Initial colonization of SL1344 purA ssaGH after infection with 8.5×10^5^ CFU. Statistical significance of colonization level differences at day 2 and 4 (for data in A), clearance at day 7 compared to day 1 (for data in B), or colonization levels at 24 h compared to 2 h (for data in C) were determined by t-test of log-transformed data (***, P<0.001; **, P<0.01; *, P<0.05). (TIF) [file pone.0042007.s001.tif]
